# Machine learning can accurately predict pre-admission baseline hemoglobin and creatinine in intensive care patients

**DOI:** 10.1038/s41746-019-0192-z

**Published:** 2019-11-29

**Authors:** Antonin Dauvin, Carolina Donado, Patrik Bachtiger, Ke-Chun Huang, Christopher Martin Sauer, Daniele Ramazzotti, Matteo Bonvini, Leo Anthony Celi, Molly J. Douglas

**Affiliations:** 1 Massachusetts Institute of Technology, Cambridge, MA USA; 2 Department of Applied Mathematics, Ecole Polytechnique, Palaiseau, France; 3 Harvard Medical School, Boston, MA USA; 4 Harvard T.H. Chan School of Public Health, Boston, MA USA; 5 Department of Pathology, Stanford University, Stanford, CA USA; 6 Department of Statistics & Data Science, Carnegie Mellon University, Pennsylvania, USA; 7 Beth Israel Deaconess Medical Center, Boston, MA USA; 8 University of Arizona College of Medicine, Tucson, AZ USA

**Keywords:** Anaemia, Acute kidney injury, Chronic kidney disease, Computational models, Data integration

## Abstract

Patients admitted to the intensive care unit frequently have anemia and impaired renal function, but often lack historical blood results to contextualize the acuteness of these findings. Using data available within two hours of ICU admission, we developed machine learning models that accurately (AUC 0.86–0.89) classify an individual patient’s baseline hemoglobin and creatinine levels. Compared to assuming the baseline to be the same as the admission lab value, machine learning performed significantly better at classifying acute kidney injury regardless of initial creatinine value, and significantly better at predicting baseline hemoglobin value in patients with admission hemoglobin of <10 g/dl.

## Introduction

Any clinical measurement is more meaningful as part of a trend over time than as a standalone data point. However, at the time of intensive care unit (ICU) admission for an acute illness, pre-admission data to inform both clinical decisions and predictive models is often unavailable. This may be due to a lack of recently drawn laboratory blood results, often compounded by poor data sharing between healthcare providers and their disparate systems.^1,2^ Clinical teams frequently make rapid, best-guess assessments of the chronicity of abnormalities, and in the setting of the clinical story, these assumptions help to guide the type and intensity of treatment. Thus far, technological advances have failed to ameliorate the subjectivity of this method of decision making.

Two frequently deranged and clinically-important laboratory values are hemoglobin and creatinine. While anemia is defined by World Health Organization as a hemoglobin value of <12 g/dl in adult women and <13 g/dl in adult men,^3^ a recent review showed that the average hemoglobin at the time of intensive care unit admission is approximately 10 g/dl.^4^ The standard of care for the past two decades has been transfusion to maintain a hemoglobin level of at least 7 g/dl.^5^ However, given that chronic anemia is generally better tolerated than acute anemia, and that transfusion itself carries risks,^6^ establishing early in the hospital stay that anemia is chronic may help to limit unnecessary transfusions.

Much like hemoglobin, creatinine levels are commonly deranged in acutely ill patients.^4^ Acute kidney injury (AKI) is defined as a creatinine increase of at least 1.5 times baseline over the last seven days or an absolute increase of ≥0.3 mg/dl over 48 h.^7^ This is in contrast to chronic kidney disease (CKD), in which derangements must persist for three months or more.^8^ By definition, this distinction relies on historical results, which may not be available. Detection of AKI will prompt a search for causes as well as the taking of corrective action, which may include fluid resuscitation and renal replacement therapy. In contrast, stable chronic kidney disease does not mandate aggressive treatment. Thus, decision making may be encumbered by lack of historical data.

Machine learning, a branch of artificial intelligence that allows computers to perform pattern recognition on datasets, is increasingly applicable in medical contexts.^9^ The ability of algorithms to extract patterns which may be non-obvious to the human observer has already been leveraged for a wide variety of future-oriented predictions including clinical deterioration,^10–13^ readmission risk,^14^ cancer prognostication,^15^ anticipation of fluid requirements in pressor-dependent patients,^16^ prediction of meaningful changes in laboratory results in the ICU,^17^ arrhythmia identification,^18^ and enhanced interpretation of medical imaging.^19–21^ Such efforts have addressed the challenge of predicting the next value or event in patients who are already in a monitored setting. However, studies seeking to quantify a “past state,” including the presence of abnormality on historical blood results, have yet to be widely attempted.

The aim of this study is to predict the prior-to-admission baseline hemoglobin and creatinine values for patients admitted to the ICU, using objective parameters available within two hours of ICU admission. We present a machine learning workflow and measures of accuracy for the models constructed to create this prediction. We contrast the interpretability of different algorithms, given that model complexity and lack of transparency may result in biased or illogical conclusions going unseen.^22^

## Results

### Cohort characteristics

The full hemoglobin and creatinine cohorts meeting inclusion criteria totaled 6139 and 4643 respectively, with 4331 patients appearing in both cohorts. The comparison cohort for which baseline lab data was not available totaled 13,853 patients. With respect to creatinine, the cohort with outpatient labs had slightly higher average age (64 vs 61) and the same median admission creatinine (0.9 mg/dl), but lower in-hospital mortality (10.4% vs 13.6%) and slightly shorter average hospital length of stay (10.5 vs 10.7 days) (Table 1). Relative to the comparison cohort, the hemoglobin cohort also had slightly higher average age (63 vs 61) and similar admission hemoglobin (11.5 vs 11.4 g/dl), but lower in-hospital mortality (12.7% vs 13.6%) and slightly shorter average length of stay (10.5 vs 10.7 days). The mean time from baseline lab draw to admission was 14.3 days for hemoglobin and 13.7 days for creatinine.

**Table 1:** Cohort characteristics.

Characteristic	Known hemoglobin baseline (*n* = 6139)	Known creatinine baseline (*n* = 4643)	Unknown outpatient baselines (*n* = 13,551)
Initial admission hemoglobin (g/dl) – Mean (interquartile range)	11.5 (10.0–13.0)	10.4 (9.0–11.5)	11.4 (9.9–13.0)
Outpatient baseline hemoglobin (g/dl) – Mean (interquartile range)	12.3 (10.8–13.7)	N/a	N/a
Initial admission creatinine (mg/dl) – Median (interquartile range)	1.0 (0.7–1.4)	0.9 (0.7–1.3)	0.9 (0.7–1.3)
Outpatient baseline creatinine (mg/dl) – Median (interquartile range)	N/a	1.29 (0.8–1.2)	N/a
Time lag from baseline lab draw to admission (days) – Mean (interquartile range)	14.3 (6–16)	13.7 (6–16)	N/a
Age – Mean (interquartile range)	63 (54–75)	64 (55–75)	61 (50–76)
ICU length of stay (hours) – Mean (interquartile range)	87 (28–89)	91 (28–94)	115 (32–121)
Hospital length of stay (days) – Mean (interquartile range)	10.5 (4–13)	10.5 (5–12)	10.7 (4–13)
In-hospital mortality (%)	12.7%	10.4%	13.6%
Gender Female (%)	42.1%	42.5%	41.6%
Vasopressors used (%)	25.8%	26%	16.7%
Admission type			
Emergency (%)	52.2%	53.0%	87.1%
Elective (%)	46.2%	45.0%	9.3%
Urgent (%)	1.6%	2.0%	3.6%

**Fig. 1:**
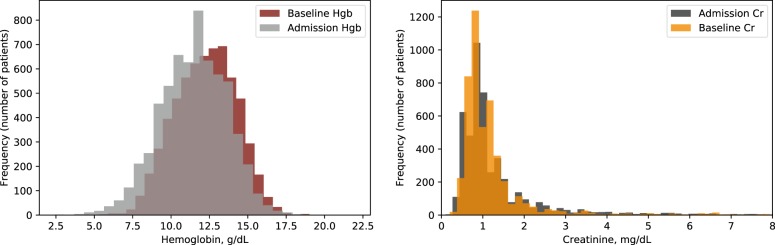
Distributions of the baseline (outpatient) and initial (admission) values for hemoglobin and creatinine. Left: Hemoglobin, Right: Creatinine.

The distributions of the baseline and initial values for hemoglobin and creatinine were overlapping (Fig. 1), but 12% of patients in each cohort showed a change in category (to hemoglobin <10 g/dl or creatinine >1.3 mg/dl) between baseline and admission.

### Primary outcome – classification task

The three best-performing models on the classification task—gradient boosted trees, random forest, and logistic regression—did not differ significantly in performance, and demonstrated areas under the receiver operating characteristic curve (AUC) of 0.86–0.89 (95% CI 0.84–0.90 across all models). Optimal classification trees (OCT) showed similar performance for hemoglobin with AUC of 0.88 (95% CI 0.87–0.89) and intermediate performance for creatinine with AUC of 0.77 (95% CI 0.76–0.79). Using a target of hemoglobin of <8 g/dl as a prediction target yielded similar AUCs to targeting hemoglobin <10 g/dl for the three top performing models, but reduced performance for OCT, with mean AUC 0.62 rather than 0.88. The legacy algorithm CART had an AUC of only 0.64 (95% CI 0.64–0.68). For creatinine, all models performed significantly better than simply assuming the baseline to be similar to the admission value, which gave an AUC of 0.5. For hemoglobin, however, assuming the baseline hemoglobin was in the same category as the admission value had an AUC of 0.78 (95% CI 0.76–0.80).

**Fig. 2:**
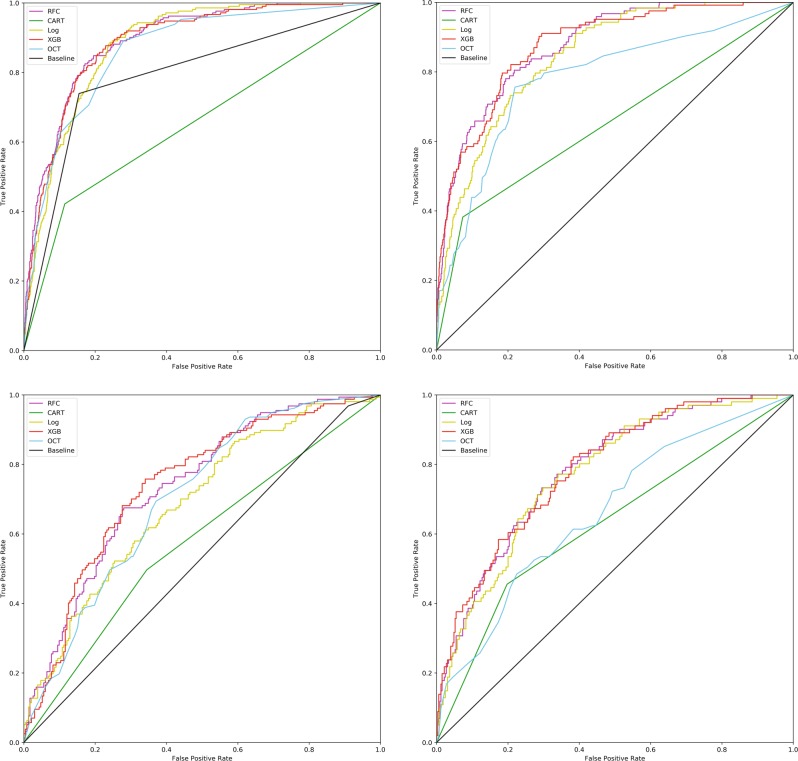
Receiver operating characteristic curves for the binary classification task by model. The left panels show performance on classifying baseline hemoglobin as <10 g/dl or not, and the right panels show performance on classifying AKI as present or absent. Upper panels show results for the full cohort, and bottom panels show results for the just the cohorts with admission hemoglobin <10 g/dl (left) and admission creatinine >1.3 mg/dl (right). The “baseline” model, shown for comparison, simply assumes the baseline value is similar to the admission value. *RFC* random forest classifier, *CART* classification and regression trees, *Log* logistic regression, *XGB* gradient boosted trees, *OCT* optimal classification trees.

Limiting the cohort to just patients with admission values for hemoglobin and creatinine in the abnormal category resulted in reduced machine learning model performance, with mean AUCs ranging from 0.66 to 0.80. The AUC for the “simple model” of assuming no change from admission value was reduced for hemoglobin and improved for creatinine with this cohort (Table 2, Fig. 2). The machine learning models’ superior performance compared to the “simple model” for both full and limited cohorts is also evident when examining precision-recall curves (Supplementary Figs 3–6), which take into account for the prevalence of true positives and true negatives in the dataset.

**Table 2:** Summary of models’ performance on classifying baseline hemoglobin as <10 g/dl or not, <8 or not, and whether AKI is present or not.

Model	AUC for baseline Hgb <10 g/dl *mean (95% CI)*		AUC for baseline Hgb <8 g/dl *mean (95% CI)*	AUC for AKI *mean (95% CI)*	
	Full Cohort *n* = 6139	Admission Hgb <10 g/dl *n* = 1553	Full Cohort *n* = 6139	Full Cohort *n* = 4643	Admission Cr >1.3 g/dl *n* = 1719
Gradient Boost	0.89 (0.88–0.90)	0.74 (0.71–0.76)	0.85 (0.81–0.88)	0.88 (0.87–0.89)	0.80 (0.76–0.82)
Random Forest	0.89 (0.88–0.90)	0.74 (0.71–0.76)	0.86 (0.81–0.92)	0.87 (0.86–0.89)	0.80 (0.76–0.83)
Logistic Regression	0.88 (0.87–0.89)	0.69 (0.67–0.72)	0.89 (0.85–0.93)	0.86 (0.84–0.88)	0.78 (0.75–0.81)
OCT (Optimal Classification Trees)	0.88 (0.87–0.89)	0.66 (0.62–0.69)	0.62 (0.53–0.71)	0.77 (0.76–0.79)	0.67 (0.65–0.70)
CART	0.66 (0.64–0.68)	0.59 (0.57–0.51)	0.57 (0.53–0.60)	0.64 (0.62–0.66)	0.61 (0.58–0.63)
Assume same as admission	0.78 (0.76–0.80)	0.53 (0.52–0.54)	0.72 (0.67–0.78)	0.5 (0.5–0.5)	0.5 (0.5–0.5)

*AUC* area under the receiver operating characteristic curve, *Hgb* hemoglobin, *AKI* acute kidney injury, *CART* Classification and Regression Trees

### Secondary outcome - continuous prediction

**Fig. 3:**
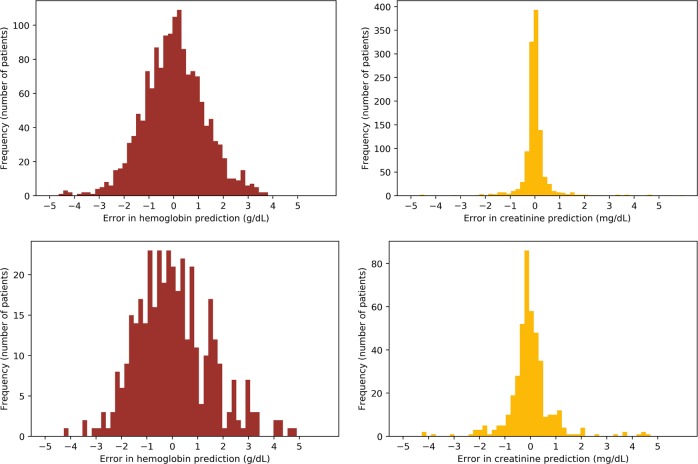
Histograms of the differences between predicted and observed baseline hemoglobin and creatinine values for the Gradient Boosted Tree model. Left panels show hemoglobin results and right panels show creatinine results. The upper panels show results for the full cohort, and bottom panels show results for the just the cohorts with abnormal admission labs – hemoglobin <10 g/dl (left) and admission creatinine >1.3 mg/dl (right).

For the continuous prediction task, i.e., predicting the actual baseline value, performance on hemoglobin prediction for the full cohort was similar across the models with mean absolute error (MAE) 0.97–1.1 g/dl. The 95% confidence interval (CI) for the best performing model, gradient boosted trees, was 0.96–0.98 g/dl. For creatinine prediction on the full cohort, MAE across the models was 0.32–0.42 mg/dl, and 95% CI for the best-performing model, gradient boosted trees, was 0.31–0.33 mg/dl. The “simple model” of just predicting the same value as admission yielded mean errors of 1.3 g/dl for hemoglobin and 0.38 mg/dl for creatinine. The MAE in all methods of prediction increased when examining just the cohort with abnormal admission values, to 1.1–1.3 g/dl (95% CI 1.1–1.3 across all models) for hemoglobin and 0.61–0.72 mg/dl (95% CI 0.59–0.77 across all models) for creatinine (Table 3, Fig. 3). Simply predicting the same value as admission for these sub-cohorts yielded mean absolute errors of 2.1 g/dl for hemoglobin and 0.75 mg/dl for creatinine.

**Table 3:** Summary of models’ performance on predicting the actual baseline value for hemoglobin and creatinine.

Model	Mean absolute error for baseline hemoglobin – g/dl *(95% CI)*		Mean absolute error for baseline creatinine – mg/dl *(95% CI)*	
	Full Cohort *n* = 6139	Cohort with Admission Hgb < 10 g/dl *n* = 1553	Full Cohort *n* = 4643	Cohort with Admission Cr > 1.3 mg/dl *n* = 6139
Gradient Boost	0.97 (0.96–0.98)	1.1 (1.1–1.1)	0.32 (0.31–0.33)	0.61 (0.59–0.63)
Random Forest	0.98 (0.96–0.99)	1.1 (1.1–1.2)	0.34 (0.33–0.35)	0.65 (0.63–0.66)
Linear Regression	1.0 (1.0–1.0)	1.2 (1.2–1.3)	0.37 (0.36–0.38)	0.70 (0.68–0.72)
ORT (Optimal Regression Trees)	1.1 (1.0–1.1)	1.3 (1.2–1.3)	0.42 (0.36–0.49)	0.72 (0.67–0.77)
Assume same as admission (“Simple model”)	1.3 (1.3–1.3)	2.1 (2.0–2.2)	0.38 (0.36–0.39)	0.75 (0.71–0.78)

*Hgb* hemoglobin, *Cr* creatinine

### Secondary outcome - model interpretability

**Fig. 4:**
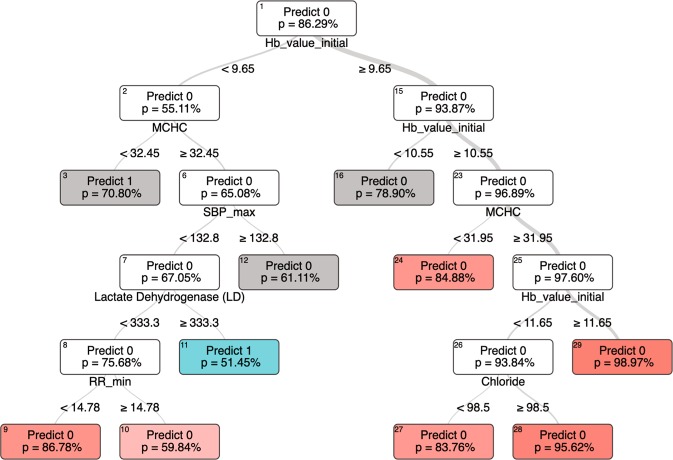
Representative optimal classification tree for the hemoglobin classification task. In this tree, “Predict 0” indicates the model’s prediction that the baseline hemoglobin is <10 g/dl, and “Predict 1” indicates a prediction that the baseline hemoglobin is 10 g/dl or greater. Relevant features generate branch points, and each terminal node (or “leaf”) represents the final model prediction. The tree shown is a segment of a larger, more complex tree diagram. Terminal nodes are colored red or green, and nodes that have additional branchings in the full model are gray. The relative thickness of the lines connecting the nodes is proportional to the fraction of patients falling on either side of the split. The “*p* value” is the model’s certainty that the categorization is correct, eg “There is a 95% chance that the baseline hemoglobin is <10 g/dl.” *Hb* hemoglobin (g/dl), *MCHC* mean corpuscular hemoglobin concentration (g/dl), *SBP* systolic blood pressure (mmHg), chloride (mEq/L), *RR* respiratory rate (breaths per minute), *max* maximum, *min* minimum.

**Fig. 5:**
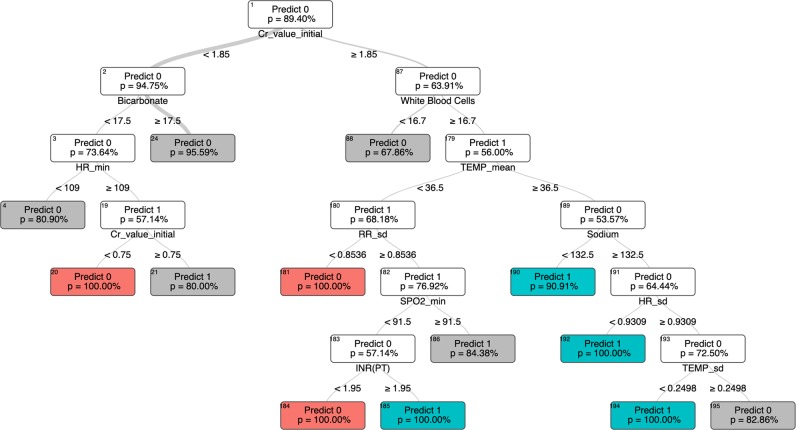
Representative optimal classification tree for the AKI classification task. “Predict 0” indicates the model’s prediction that AKI is present, and “Predict 1” indicates a prediction of no AKI. Please see full explanation of the tree diagram in Fig. 4. *Cr* creatinine (mg/dl), bicarbonate (mmol/L), white blood cells (thousand/μL), *HR* Heart Rate (beats per minute), *TEMP* temperature (degrees celsius), *RR* respiratory rate (breaths per minute), sodium (mEq/dl), *SPO2* oxygen saturation (%), *INR(PT)* international normalized ratio of prothrombin time, *mean* average, *min* minimum, *sd* standard deviation.

Model interpretability is optimized when a human being can understand how a computer model arrived at a particular conclusion. Of the models tested, Optimal Classification Trees is qualitatively the most interpretable, as the entire model can be represented visually as a single branching decision tree, as shown in Figs 4 and 5.^23,24^ Ensemble methodologies like random forest^25^ and gradient boosted trees^26^ produce multiple decision trees, each built from a subset of the data, that are then combined to yield the model output. However, these “forests” of decision trees do not lend themselves to a cohesive visual representation and are not shown.

Assessment of feature importance was also done for all models. The admission value of the lab test of interest was consistently the most important model input. For hemoglobin, the mean corpuscular hemoglobin concentration was reliably the next second most important feature (Supplementary Figs 15–19). For creatinine and AKI prediction, the other parameters consistently selected as important were blood urea nitrogen, electrolytes, age, heart rate and blood pressure (Supplementary Figs 20–24).

## Discussion

It is common for patients presenting with acute illness to have multiple derangements in vital signs and laboratory results, without clarity as to which abnormalities are due to acute illness versus chronic comorbidities. In the absence of historical data, clinical teams use their judgement to estimate the chronicity of abnormalities; these assumptions often guide the type and intensity of treatment. The goal of this work is to improve clinical care through the accurate back-prediction of the pre-admission baseline hemoglobin and creatinine using data commonly available within the first several hours of ICU stay.

We trained and tested several prediction methods, including logistic and linear regression, gradient boosted trees, random forest, and optimal trees. These models performed well on the classification task with AUC’s of 0.86–0.89. The regression models demonstrated the ability to predict hemoglobin and creatinine to within clinically-informative ranges, averaging within 0.97–1.1 g/dl of the true value for hemoglobin and 0.32–0.42 mg/dl for creatinine. As the inter-analyzer laboratory variability for hemoglobin measurement has been reported at 0.3–1.5 g/dl,^27^ and for creatinine at 0.1–0.2 mg/dl,^28^ model precision approaches that of existing laboratory testing on varied equipment. Compared to assuming the baseline to be the same as the admission lab value, machine learning performed significantly better at classifying acute kidney injury regardless of initial creatinine value, and significantly better at predicting baseline hemoglobin value in patients with admission hemoglobin of <10 g/dl. On other outcome measures, i.e. classifying baseline hemoglobin as greater or less than <10 g/dl regardless of admission value, or predicting actual baseline creatinine, the “simple model” of assuming that the baseline is the same as the initial lab value performed nearly as well. Thus, machine learning can lend precision to the baseline lab prediction, and whether or not this affects treatment decisions will depend on individual patient context.

Overall, model performance on the subsets of patients with abnormal admission labs was decreased compared to that on the full cohorts (Tables 2 and 3). The sample sizes for these subgroups were only 26–37% of the size of the full cohorts, therefore there were fewer data points for the algorithm to learn from and performance may have been decreased in part for this reason.

We explored this further with Bland–Altman plots (see Supplementary Information). For all hemoglobin prediction models, there was a tendency for the model to under-predict high baseline levels and over-predict if the true baseline was low. We suspect this is related to having relatively few patients to learn from at the extremes of the hemoglobin range, eg. hemoglobin <9 or >13 g/dl (Fig. 1, Supplementary Figs 7–10). For creatinine, there was also a trend to under-predict high baseline values, but this was most pronounced at levels of creatinine consistent with renal failure, i.e., >4–6 mg/dl (Supplementary Figs 11–14). This may be partly related to the fact that for chronic renal failure patients, a creatinine of 4 g/dl and 6 g/dl may occur within the same week (as the value varies with dialysis timing) without significant vital sign changes to give the algorithm a reason to predict a higher value.

The features identified by the models as important predictors of baseline hemoglobin and creatinine levels appear clinically appropriate. For both, the admission value was the strongest predictor of the baseline value. For hemoglobin, MCHC, a measure of the concentration of hemoglobin in a given volume of packed red blood cells, tends to be decreased in chronic but not acute anemia,^29^ and this was reflected in the model. For creatinine, BUN as well as other electrolytes that are handled by the kidneys affected prediction of recent changes in renal function, as did more advanced age. Vital signs including heart rate and blood pressure were important features in the models, as they may be affected by anemia and may also suggest changes in end organ perfusion that may be causative of acute kidney injury. While previous work has shown that both random forest models as well as multiple imputation with chained equations (MICE)^30,31^ perform well for the imputation of missing laboratory values, our work builds on this by adding the rich clinical information available from vital signs to help with imputing a value more distant in time, the pre-illness baseline.

Non-intuitive interactions were also identified in the optimal classification trees. For instance in one of the generated trees (Supplementary Fig. 25), glucose >450 mg/dl was predictive of normal renal function. Upon further investigation, a bimodal age distribution of the patients with glucose >450 mg/dl was noted (see Supplementary Figs 26 and 27), suggesting that in some cases a markedly elevated glucose was simply a marker of younger age, which tends to be associated with better renal function. We suspect this finding was produced by a cohort of otherwise-healthy younger patients admitted for uncontrolled type I diabetes.

The capacity for model interpretability can help to address valid concerns around bias and discrimination in datasets, and how these may have unseen impacts on the output of uninterpretable machine learning models.^32,33^ We hypothesize that this interpretability may ultimately increase the acceptance and clinical utility of machine learning, by allowing providers to understand and gain trust in the models’ methodology and outputs. While this work focused on information from a de-identified and structured clinical database, it uses values that are commonly charted. Deploying an algorithm within the EHR, in which the record might report “predicted” as well as “measured” laboratory values, is a future possibility. Further, existing illness severity scores, e.g., SOFA^34^ or APACHE II,^35^ operate on the premise that the patient’s biomarkers were normal prior to illness. This does not allow the score to distinguish chronic comorbidities from acute derangements. The ability to impute a “well” baseline that is individualized to a given patient would permit more nuanced categorization, for instance creating different renal function scores for a young patient with AKI versus an elderly patient with chronic kidney disease. For critical care research where many physiologic derangements coexist, this may be particularly useful in identifying subgroups of patients who benefit from various treatments.

Algorithm deployment within an EHR would face validation, regulatory, and privacy challenges.^36^ Notably, as machine learning algorithms are trained to predict outcomes based on past events, they run the risk of perpetuating biases rather than promoting objectivity. Rigorous comparisons of model classification for privileged vs at-risk subgroups has been described.^37^ Attention to these details will be important future work for any machine learning deployed in the healthcare setting, where bias is well known to affect patient-health system interactions.^38,39^

This study has several limitations. Only patients with prior-to-admission labs were included, potentially yielding a sicker cohort compared to patients without these outpatient healthcare contacts, which may limit generalizability. However, the shorter length of stay and lower mortality in the group with outpatient lab draws argues against this cohort being significantly sicker. The fact that MIMIC-III is a single-center database also limits external validity of the results. Our machine learning workflow for predicting baseline labs, however, may be adapted for other institutions, allowing the re-training of models to fit different populations.

This work assumes a state of stability or wellness when labs are drawn in the outpatient setting. Although the state of health required to present for outpatient blood test versus that requiring admission to the ICU are highly divergent, the former may not represent a “stable” baseline for all patients.

We chose two hours of ICU time as a clinically reasonable cut point for data capture prior to model training and testing. This is a trade-off, as longer data-capture could produce a more accurate prediction, but the clinical utility of the prediction wanes the longer it is delayed. Further, as MIMIC-III is an ICU database, we were not able to incorporate vital sign data from the emergency department or non-ICU wards. If such data were available, it might allow predictions of similar accuracy to be made at an earlier time point.

In summary, the use of statistical and machine learning models enables accurate prediction of the prior-to-admission baseline hemoglobin and creatinine levels, using data available within two hours of ICU admission. Compared to assuming the baseline to be the same as the admission lab value, machine learning performed significantly better on classifying acute kidney injury regardless of initial creatinine value, and significantly better at predicting baseline hemoglobin value in patients with admission hemoglobin of <10 g/dl.

## Methods

This study is reported in accordance with the STrengthening the Reporting of OBservational studies in Epidemiology (STROBE) statement.^40^ The project was approved by the Institutional Review Board of the Beth Israel Deaconess Medical Center (IRB Protocol #2001P001699) and was granted a waiver of informed consent.

We used retrospective data from the Medical Information Mart for Intensive Care (MIMIC-III, version 1.4) database; a single center, publically available, de-identified high-resolution database of ICU stays, built and maintained by the Laboratory for Computational Physiology at Massachusetts Institute of Technology (MIT). MIMIC-III is available at http://mimic.physionet.org/. MIMIC-III includes 46,520 patients with intensive care unit admissions between 2001 and 2012 at Beth Israel Deaconess Medical Center in Boston.^41^ While the database is comprehensive for ICU stays, including bedside monitor vital sign data, laboratory values, and full text of chart notes, it also includes outpatient laboratory results when samples were processed within the Beth Israel Deaconess system.

### Derivation and validation cohorts

**Fig. 6:**
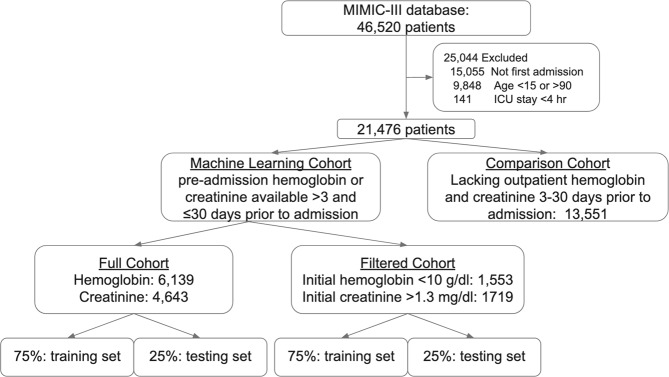
Cohort selection process from the MIMIC-III database.

Using the MIMIC-III database, we identified patients aged 15 to 90 admitted to intensive care who also had prior-to-admission blood tests drawn for hemoglobin or creatinine in the outpatient setting between three and 30 days prior to admission. The outpatient setting was used as a proxy for the patient being in a well state, and the prior to admission value was taken to represent the patient’s recent “baseline”. Ninety was chosen as an age cut-off, as patients older than 90 have their ages masked for confidentiality in MIMIC-III. For patients with more than one ICU stay, only the first ICU stay was used. Patients were excluded if the ICU stay was shorter than four hours, and if they did not have the lab of interest (hemoglobin or creatinine) tested within two hours before or after ICU admission. Two final cohorts of 6139 and 4643 patients were identified for the hemoglobin and creatinine prediction tasks respectively (Fig. 6). As hemoglobin and creatinine are often tested together, 4331 patients appeared in both groups. In total 75% of each cohort was used for model training. The remaining 25% was used for validation and testing, and all reported metrics of model performance were obtained from this subset. The cohort of patients meeting all inclusion criteria except for the availability of an outpatient lab measurement was also extracted, for comparison of baseline characteristics (Table 1).

### Independent variables and outcomes

The primary outcome measure was model performance on a binary classification task. For hemoglobin, we predicted whether the baseline hemoglobin was <10 g/dl or not, in accordance with the World Health Organization cut-off for moderate anemia.^42^ The creatinine binary prediction target was the presence or absence of acute kidney injury, i.e., whether the admission creatinine relative to the predicted baseline showed an increase of 1.5 fold, in accordance with KDIGO guidelines.^7^ Given that patients presenting with normal hemoglobin and creatinine levels were highly likely to have normal baselines, the primary outcomes were also assessed on the subset of patients presenting with hemoglobin of <10 g/dl or creatinine >1.3 mg/dl. This cohort was significantly smaller, totaling 1553 patients for hemoglobin and 1719 for creatinine. For hemoglobin, we additionally tested model performance for identifying baseline hemoglobin of <8 g/dl, as this is closer to most clinicians’ transfusion threshold.

Secondary outcomes were model performance on a regression task in which the actual baseline value was predicted for hemoglobin and creatinine, and a qualitative assessment of model interpretability.

The independent variables selected from MIMIC-III and used as model inputs are shown in full in Table 4, and included patient demographics such as age, gender, vital signs (temperature, heart rate, blood pressure, respiratory rate and oxygen saturation), and laboratory values including complete blood count, red cell indices, electrolytes, coagulation studies, and renal and liver function tests as available within two hours of ICU admission. Time-series vital signs were summarized as maximum, minimum, mean, and standard deviation over the two hour period. Data extraction and preprocessing was accomplished using R (R Foundation for Statistical Computing, Vienna, Austria, v3.5.0),^43^ the Google BigQuery application programming interface (Google LLC, Mountain View, CA),^44^ and the community developed IPython package Pandas^45^ (v0.23.4).

**Table 4:** Demographic, vital sign, and laboratory data extracted as model inputs.

Demographics	Vital signs	Laboratory values	
Age at admission	Temperature	Hemoglobin	Bilirubin
Sex	Heart rate	White blood cell count (WBC)	pH
Time from baseline lab draw to admission	Systolic blood pressure	Platelet count	Lactate dehydrogenase (LDH)
	Diastolic blood pressure	Sodium	International normalized ratio (INR)
	Respiratory rate	Potassium	Prothrombin time (PTT)
	Oxygen saturation	Bicarbonate	Red blood cell count (RBC)
		Chloride	Mean corpuscular volume (MCV)
		Blood urea nitrogen (BUN)	Mean corpuscular hemoglobin concentration (MCHC)
		Creatinine	Iron
		Glucose	Transferrin
		Calcium	Ferritin
		Phosphate	Folate
		Albumin	B12
		Partial pressure of CO2	Lactate

### Missing data

The majority of independent variables were missing for less than 20% of patients in the creatinine cohort, and less than 30% of patients in the hemoglobin cohort (See Supplementary Figs 1 and 2). The missingness distributions were bimodal, with a second peak above 80% missing, and features with greater than 80% missing data were excluded. This resulted in the removal of seven initially-extracted features: ferritin, folate, iron, transferrin, total iron binding capacity, total protein, and vitamin B12 from the analysis. Missing data for all other features were imputed, testing both simple mean imputation and optimal *k* nearest-neighbor (K-NN) imputation.^46^ K-NN imputation, in which the imputed value is informed by the values of otherwise-similar patients, improved the models’ area under the curve by 1% or less compared to mean imputation, but was kept in the final analysis pipeline to improve future generalizability to other datasets. Specifically, imputation over clusters of patients with similar physiology may produce more physiologically accurate results. Furthermore, avoiding imputing a whole-sample mean prevents the mean value taking on unexpected significance during machine learning, as a surrogate marker of missing values.

### Modeling

We tested and compared several modeling algorithms for performance and interpretability, including the ensemble methodologies random forest,^25^ gradient boosted trees^26^ and classification and regression trees (CART),^47^ and as well as non-ensemble methods, namely optimal classification trees,^23,24^ linear regression, and holistic logistic regression.^48^ To enhance model training, a grid search pipeline was built with several train-validation-test splits to identify the best-performing hyperparameters (e.g., tree depth, number of trees, number of features considered for each split) for each model. Machine learning processes as well as missing data imputation were carried out with the use of Python v3.5.6 (The Python Software Foundation, Beaverton, OR) and Julia v6.4 (NumFOCUS, Austin, TX) programing languages. Community-developed packages including IPython,^49^ Matplotlib^50^ (v3.0), Scikit-learn^51^ (v0.20.0), SciPy^52^ (v1.1.0), Pandas^45^ (v0.23.4), as well as packages built by the Operations Research Center of MIT including OptImpute and OptimalTrees^23,24^ were used.

For the primary outcome measure of model performance on the classification tasks, models were compared on area under the receiver operating characteristic curve (ROC). To quantify uncertainty in model performance, bootstrapped cohorts were generated from random sub-samplings of the dataset, and 95% confidence intervals were calculated.

For prediction of the actual baseline lab value, performance was assessed on the mean absolute error, i.e. the average of the absolute value of the difference between the observed and predicted value.

## Supplementary information

Supplementary Information

Reproducibility Checklist

## Data Availability

Access to the MIMIC-III database may be requested via: https://mimic.physionet.org/.

## Code availability

All code generated to produce this work is available at: https://github.com/adauvin/Back-prediction

## References

[1] Deyo D, Khaliq A, Mitchell D, Hughes DR (2018). Electronic sharing of diagnostic information and patient outcomes. Am. J. Manag Care.

[2] Rudin RS, Motala A, Goldzweig CL, Shekelle PG (2014). Usage and effect of health information exchange: a systematic review. Ann. Intern. Med..

[3] World Health Organization. Haemoglobin concentrations for the diagnosis of anaemia and assessment of severity (2011).

[4] Tyler PD (2018). Assessment of intensive care unit laboratory values that differ from reference ranges and association with patient mortality and length of stay. JAMA Netw. Open.

[5] Hébert PC (1999). A multicenter, randomized, controlled clinical trial of transfusion requirements in critical care. Transfusion Requirements in Critical Care Investigators, Canadian Critical Care Trials Group. N. Engl. J. Med..

[6] Carson JL (2012). Red blood cell transfusion: a clinical practice guideline from the AABB*. Ann. Intern. Med..

[7] Khwaja A (2012). KDIGO clinical practice guidelines for acute kidney injury. Nephron Clin. Pr..

[8] Levey AS (2005). Definition and classification of chronic kidney disease: a position statement from Kidney Disease: Improving Global Outcomes (KDIGO). Kidney Int..

[9] Esteva A (2019). A guide to deep learning in healthcare. Nat. Med..

[10] Celi LAG (2011). A clinical database-driven approach to decision support: predicting mortality among patients with acute kidney injury. J. Healthc. Eng..

[11] Moreno RP (2008). Sepsis mortality prediction based on predisposition, infection and response. Intensive Care Med..

[12] Somanchi, S., Adhikari, S., Lin, A., Eneva, E. & Ghani, R. In *Proceedings of the 21th ACM SIGKDD International Conference on Knowledge Discovery and Data Mining - KDD ’15* 2119–2126 (ACM Press, 2015). 10.1145/2783258.2788588.

[13] Tomašev N (2019). A clinically applicable approach to continuous prediction of future acute kidney injury. Nature.

[14] Frizzell JD (2017). Prediction of 30-day all-cause readmissions in patients hospitalized for heart failure: comparison of machine learning and other statistical approaches. JAMA Cardiol..

[15] Kourou K, Exarchos TP, Exarchos KP, Karamouzis MV, Fotiadis DI (2015). Machine learning applications in cancer prognosis and prediction. Comput. Struct. Biotechnol. J..

[16] Celi LA, Hinske Christian L, Alterovitz G, Szolovits P (2008). An artificial intelligence tool to predict fluid requirement in the intensive care unit: a proof-of-concept study. Crit. Care.

[17] Cismondi F (2013). Reducing unnecessary lab testing in the ICU with artificial intelligence. Int J. Med Inf..

[18] Hannun AY (2019). Cardiologist-level arrhythmia detection and classification in ambulatory electrocardiograms using a deep neural network. Nat. Med..

[19] Lee J-G (2017). Deep learning in medical imaging: general overview. Korean J. Radiol..

[20] Gulshan V (2016). Development and validation of a deep learning algorithm for detection of diabetic retinopathy in retinal fundus photographs. JAMA.

[21] Wang S, Summers RM (2012). Machine learning and radiology. Med. Image Anal..

[22] Caruana, R. et al. Intelligible Models for HealthCare: Predicting Pneumonia Risk and Hospital 30-day Readmission. in *Proceedings of the 21th ACM SIGKDD International Conference on Knowledge Discovery and Data Mining* 1721–1730 (ACM, 2015). 10.1145/2783258.2788613.

[23] Bertsimas D, Dunn J (2017). Optimal classification trees. Mach. Learn..

[24] Interpretable AI, LLC. Interpretable AI Documentation (2019).

[25] Breiman L (2001). Random forests. Mach. Learn..

[26] Chen, T. & Guestrin, C. XGBoost: a scalable tree boosting system. in *Proceedings of the 22Nd ACM SIGKDD International Conference on Knowledge Discovery and Data Mining* 785–794 (ACM, 2016). 10.1145/2939672.2939785.

[27] Shah N, Osea EA, Martinez GJ (2014). Accuracy of noninvasive hemoglobin and invasive point-of-care hemoglobin testing compared with a laboratory analyzer. Int. J. Lab. Hematol..

[28] Lee E, Collier CP, White CA (2017). Interlaboratory variability in plasma creatinine measurement and the relation with estimated glomerular filtration rate and chronic kidney disease diagnosis. Clin. J. Am. Soc. Nephrol..

[29] Sarma, P. R. Red Cell Indices. in Clinical Methods: The History, Physical, and Laboratory Examinations (eds. Walker, H. K., Hall, W. D. & Hurst, J. W.) (Butterworths, 1990). 21250045

[30] Waljee Akbar K, Mukherjee Ashin, Singal Amit G, Zhang Yiwei, Warren Jeffrey, Balis Ulysses, Marrero Jorge, Zhu Ji, Higgins Peter DR (2013). Comparison of imputation methods for missing laboratory data in medicine. BMJ Open.

[31] Luo Y, Szolovits P, Dighe AS, Baron JM (2018). 3D-MICE: integration of cross-sectional and longitudinal imputation for multi-analyte longitudinal clinical data. J. Am. Med Inf. Assoc..

[32] Vayena E, Blasimme A, Cohen IG (2018). Machine learning in medicine: addressing ethical challenges. PLOS Med..

[33] Pivovarov R, Albers DJ, Sepulveda JL, Elhadad N (2014). Identifying and Mitigating Biases in EHR Laboratory Tests. J. Biomed. Inf..

[34] Vincent JL (1996). The SOFA (Sepsis-related Organ Failure Assessment) score to describe organ dysfunction/failure. On behalf of the Working Group on Sepsis-Related Problems of the European Society of Intensive Care Medicine. Intensive Care Med..

[35] Knaus WA, Draper EA, Wagner DP, Zimmerman JE (1985). APACHE II: a severity of disease classification system. Crit. Care Med..

[36] Jiang F (2017). Artificial intelligence in healthcare: past, present and future. Stroke Vasc. Neurol..

[37] Chouldechova, A. & G’Sell, M. Fairer and more accurate, but for whom? *preprint arXiv:1707.00046 [cs, stat]* (2017).

[38] Hall WJ (2015). Implicit racial/ethnic bias among health care professionals and its influence on health care outcomes: a systematic review. Am. J. Public Health.

[39] Forhan M, Salas XR (2013). Inequities in healthcare: a review of bias and discrimination in obesity treatment. Can. J. Diabetes.

[40] Vandenbroucke JP (2007). Strengthening the reporting of observational studies in epidemiology (STROBE): explanation and elaboration. Ann. Intern. Med..

[41] Johnson AEW (2016). MIMIC-III, a freely accessible critical care database. Sci. Data.

[42] DeMaeyer, E. M. et al. Preventing and Controlling Iron Deficiency Anaemia Through Primary Health Care - A guide for health administrators and programme managers. *World Health Organization - Geneva* 61 (1989).

[43] R Core Team, R. F. for S. C. R: A Language and Environment for Statistical Computing (2018).

[44] An Interface to Google’s ‘BigQuery’ ‘API’.

[45] McKinney, W. Data Structures for Statistical Computing in Python. 6 (2010).

[46] Bertsimas D, Pawlowski C, Zhuo YQ (2018). From predictive methods to missing data 563 imputation: an optimization approach. J. Mach. Learn. Res..

[47] Breiman, L., Friedman, J., Stone, C. J. & Olshen, R. A. *Classification and Regression Trees*. (Wadsworth. Republished by CRC Press, 1984).

[48] Bertsimas D, King A (2015). OR Forum—an algorithmic approach to linear regression. Oper. Res..

[49] Perez F, Granger BE (2007). IPython: a system for interactive scientific computing. Comput. Sci. Eng..

[50] Hunter JD (2007). Matplotlib: a 2D graphics environment. Comput. Sci. Eng..

[51] Pedregosa F (2011). Scikit-learn: machine learning in python. J. Mach. Learn. Res..

[52] Jones, E., Oliphant, T. & Peterson, P. SciPy: Open source scientific tools for Python. (2001). http://www.scipy.org/.

