# Supplementary material for: Machine learning can accurately predict pre-admission baseline hemoglobin and creatinine in intensive care patients
Source: NPJ Digit Med. 2019 Nov 29;2:116. doi: 10.1038/s41746-019-0192-z (PMC6884624; doi:10.1038/s41746-019-0192-z)
Supplement: Supplementary file 1 — Supplementary Information [file 41746_2019_192_MOESM1_ESM.pdf]

Supplementary Information: Machine learning can accurately predict pre-admission baseline hemoglobin and creatinine in intensive care patients

Antonin Dauvin, MS;
Carolina Donado, MD;
Patrik Bachtiger, MBBS MSc MRCP;
Ke-Chun Huang, Ph.D;
Christopher Martin Sauer, MD MPH;
Daniele Ramazzotti, PhD;
Matteo Bonvini;
Leo Anthony Celi, MD MS MPH;[*]
Molly J Douglas, MD[†]

[*]lceli@bidmc.harvard.edu
[†]mjdouglas@surgery.arizona.edu

1 Missing Data

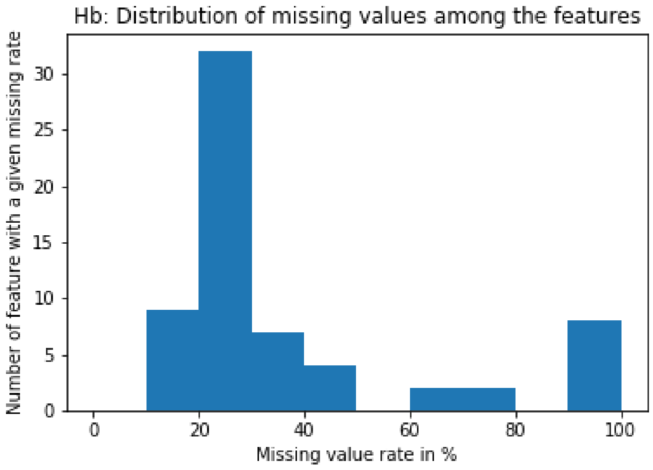

Supplementary Figure 1: Missing data distribution: Hemoglobin cohort

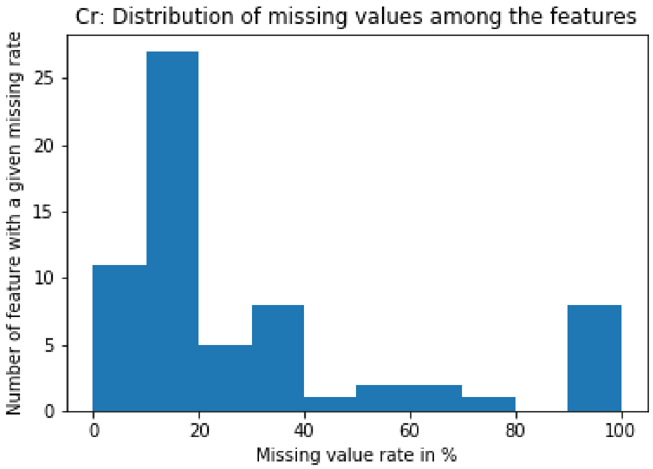

Supplementary Figure 2: Missing data distribution: Creatinine cohort

2 Precision Recall Plots - Hemoglobin classification task

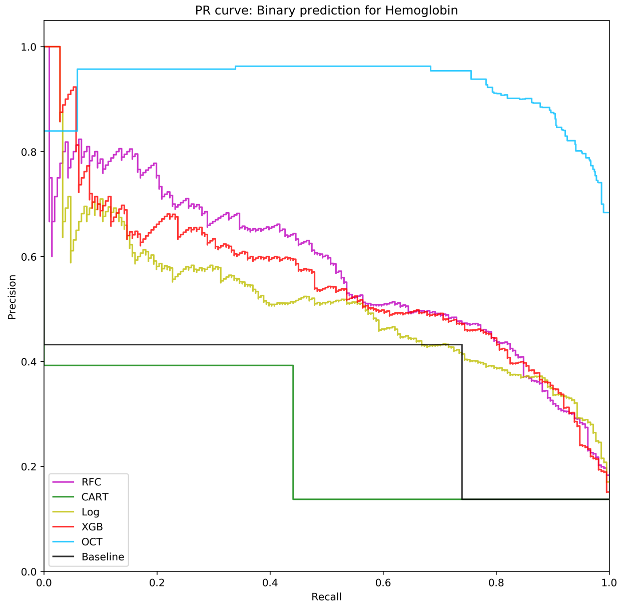

Supplementary Figure 3: **Precision Recall Plot: Full Hemoglobin Cohort**

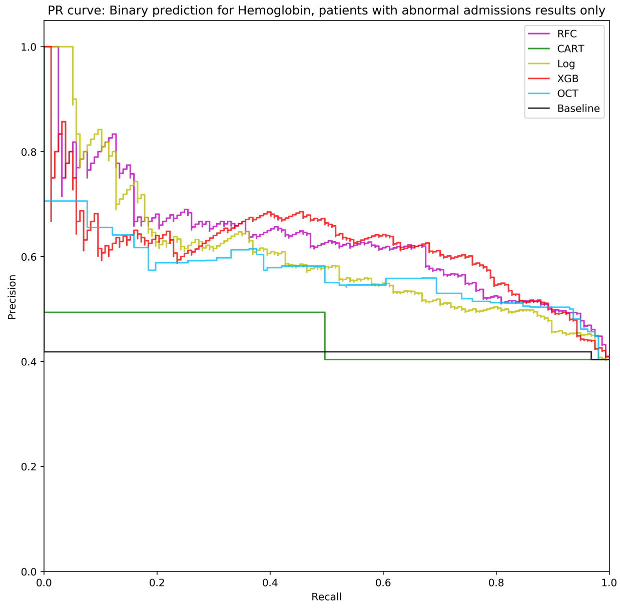

Supplementary Figure 4: **Precision Recall Plot: Cohort with admission hemoglobin <10 g/dl** (RFC - Random forest classifier, CART - classification and regression trees, Log - Logistic regression, XGB - Gradient boosted trees, OCT - optimal classification trees, "Baseline" - simple model of assuming baseline value is same as admission value.)

3 Supplementary Note 1 - Regarding hemoglobin prediction performance

We had only 341 patients with admission hemoglobin of <8 g/dl, which we did not feel was enough to include modeling based on this cohort in the main manuscript. However, given that hemoglobin <8 g/dl may be a more clinically actionable prediction target than <10 g/dl we would like to present the following: Using the gradient boosted tree model trained on the whole hemoglobin cohort, we re-assessed the MAE for continuous baseline prediction for just the cohort of patients with admission hemoglobin <8 g/dl. This yielded MAE of 1.31 (95% CI 1.29-1.35). By contrast, the "simple model" of assuming no change in hemoglobin for this cohort yielded MAE of 3.32 (95% CI 3.22 - 3.43).

4 Precision Recall Plots - AKI classification task

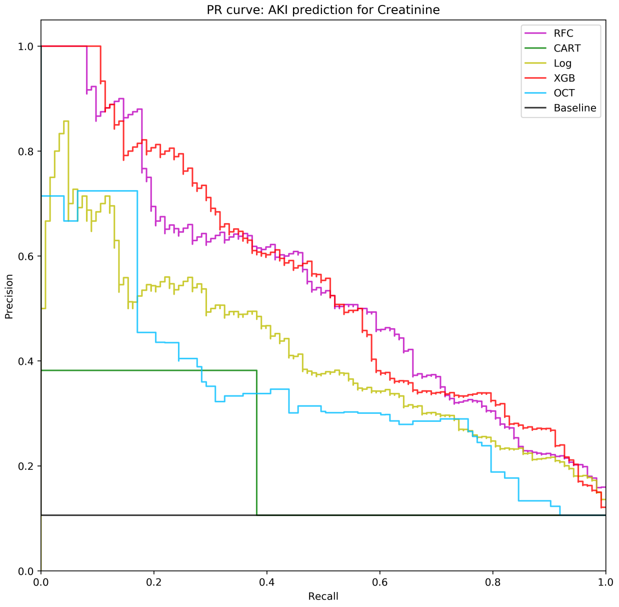

Supplementary Figure 5: **Precision Recall Plot: Full Creatinine Cohort**

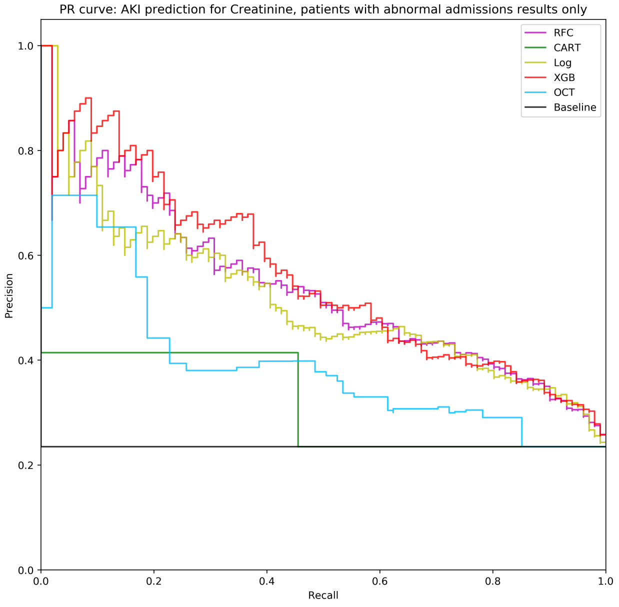

Supplementary Figure 6: **Precision Recall Plot: Cohort with admission creatinine >1.3 mg/dl** (RFC - Random forest classifier, CART - classification and regression trees, Log - Logistic regression, XGB - Gradient boosted trees, OCT - optimal classification trees, "Baseline" - simple model of assuming baseline value is same as admission value.)

5 Bland-Altman Plots by model - Hemoglobin

Error in continuous hemoglobin prediction as a function of hemoglobin baseline value

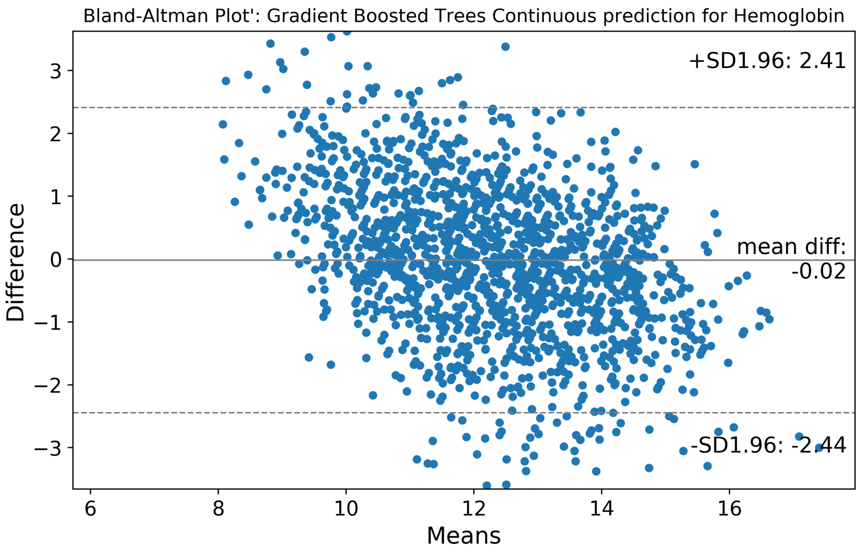

Supplementary Figure 7: Gradient Boosted Trees Bland-Altman Plot - Hemoglobin

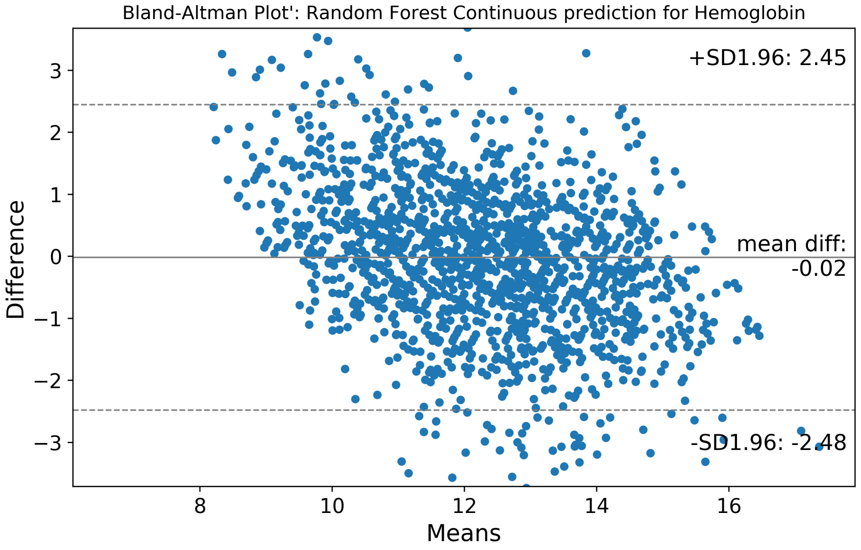

Supplementary Figure 8: Random Forest Bland-Altman Plot - Hemoglobin

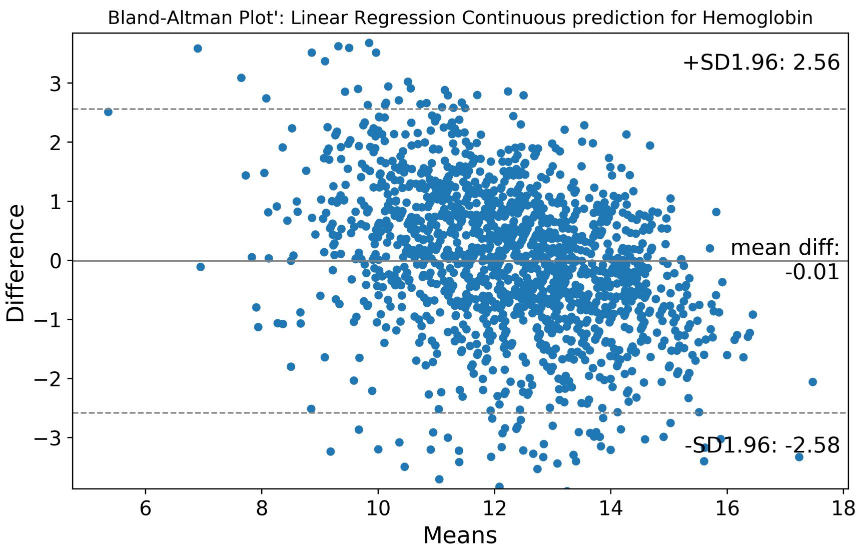

Supplementary Figure 9: Linear Regression Bland-Altman Plot - Hemoglobin

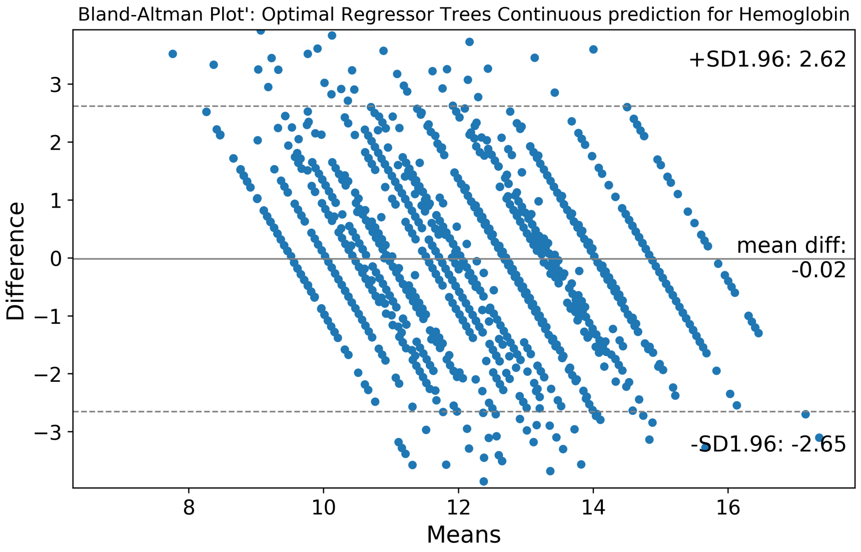

Supplementary Figure 10: Optimal Regression Trees Bland-Altman Plot - Hemoglobin

6 Bland-Altman Plots by model - Creatinine

Error in continuous creatinine prediction as a function of creatinine baseline value

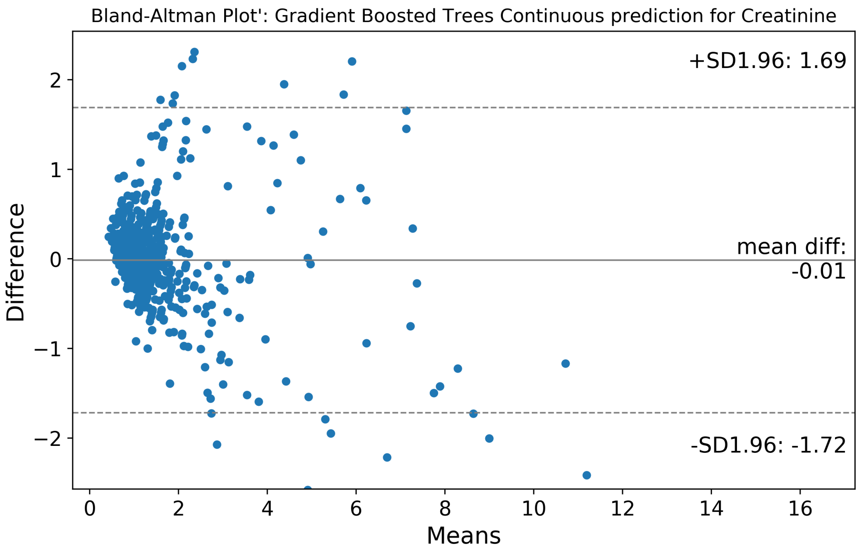

Supplementary Figure 11: Gradient Boosted Trees Bland-Altman Plot - Creatinine

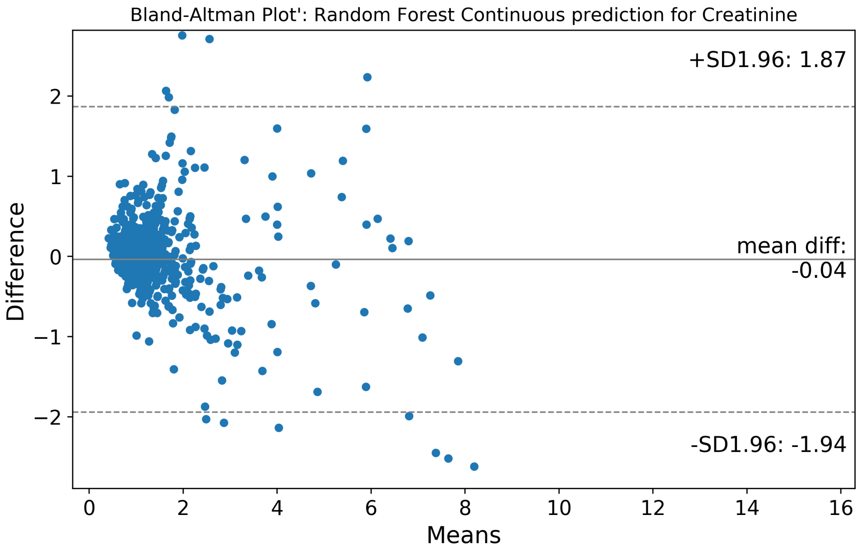

Supplementary Figure 12: Random Forest Bland-Altman Plot - Creatinine

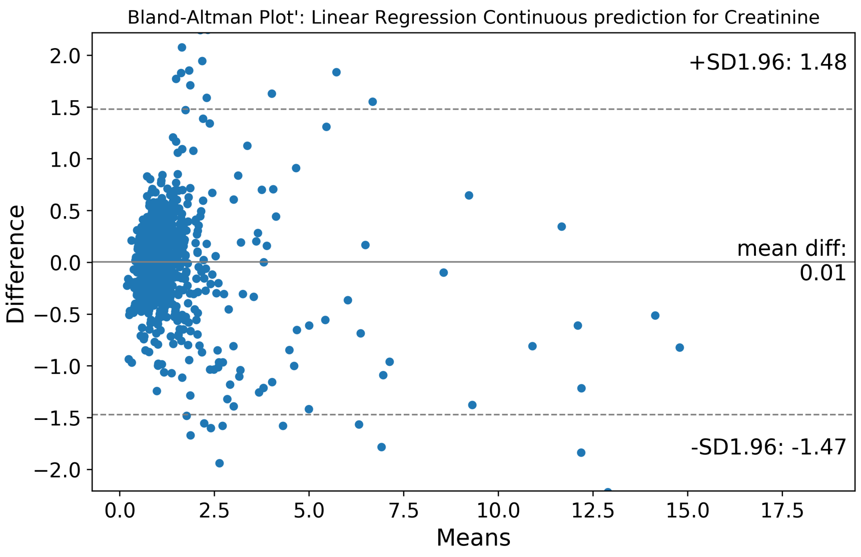

Supplementary Figure 13: Linear Regression Bland-Altman Plot - Creatinine

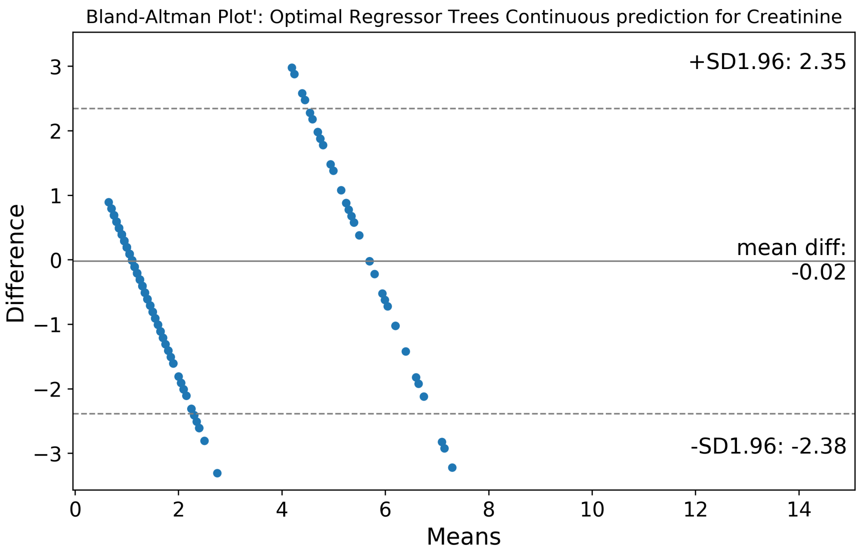

Supplementary Figure 14: Optimal Regression Trees Bland-Altman Plot - Creatinine

7 Feature Importance by model - Hemoglobin Classification Task

(Shown Feature Importances are entropy-based, with the exception of logistic regression for which model coefficients are shown)

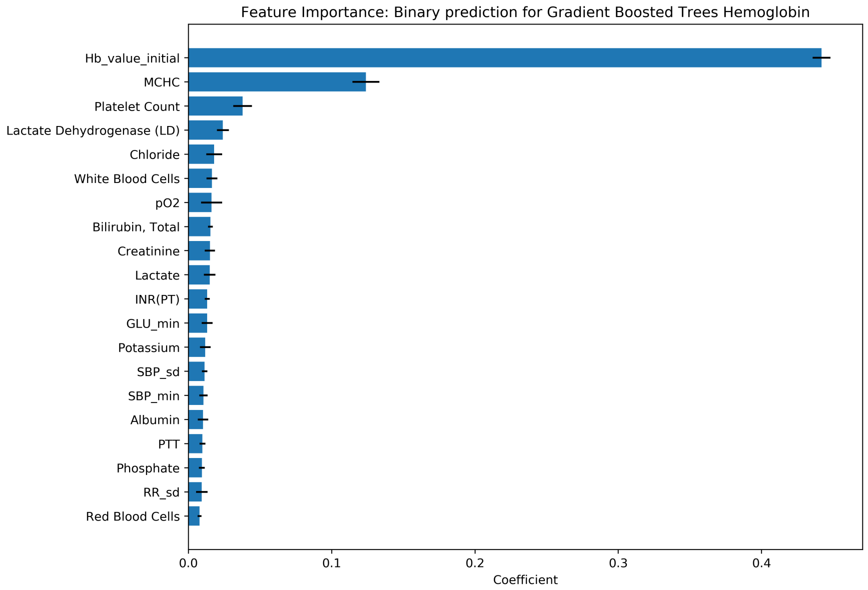

Supplementary Figure 15: **Gradient Boosted Trees Feature Importance - Hemoglobin Classification**

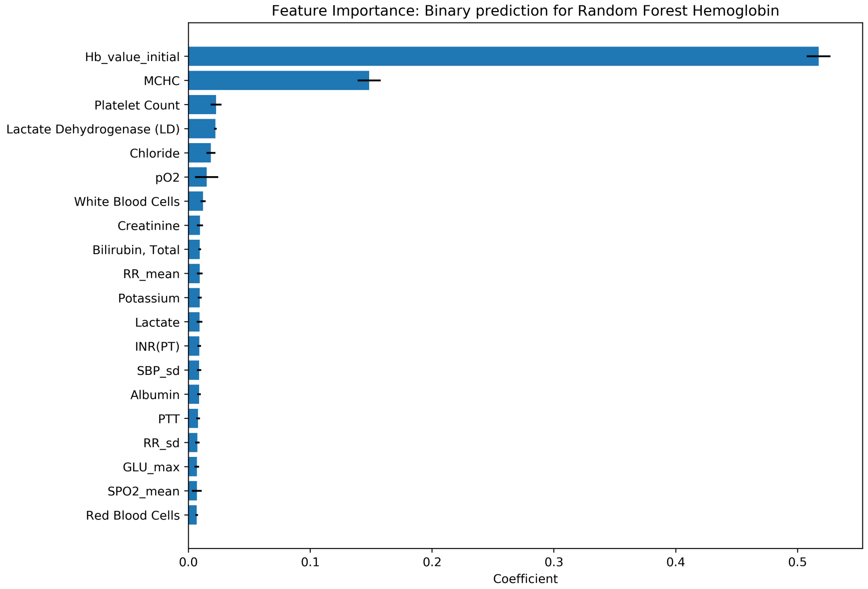

Supplementary Figure 16: **Random Forest Feature Importance - Hemoglobin Classification**

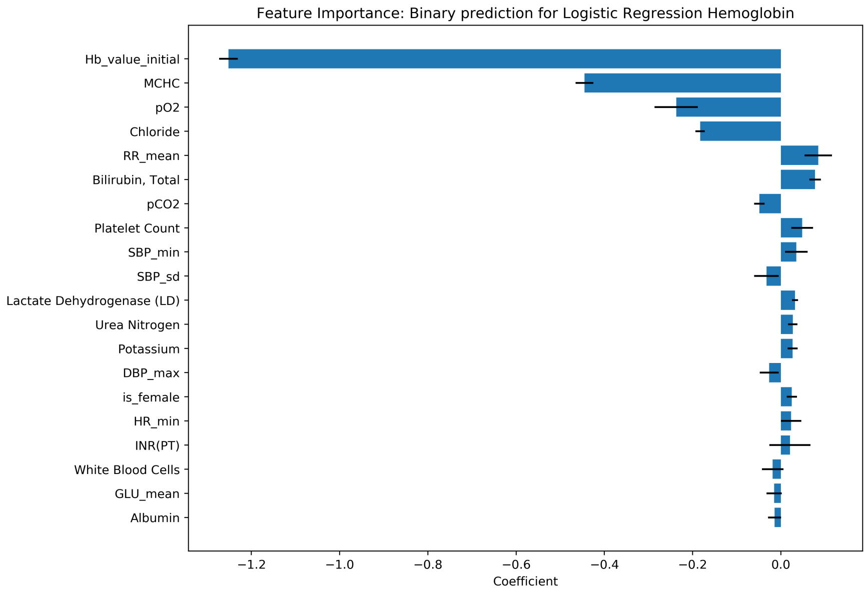

Supplementary Figure 17: **Logistic Regression Feature Importance - Hemoglobin Classification**

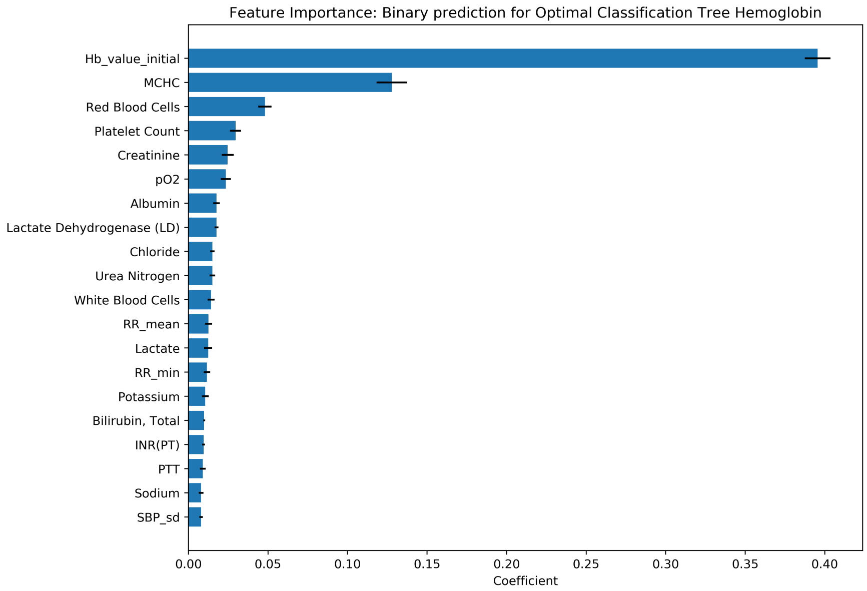

Supplementary Figure 18: **Optimal Classification Trees Feature Importance - Hemoglobin Classification**

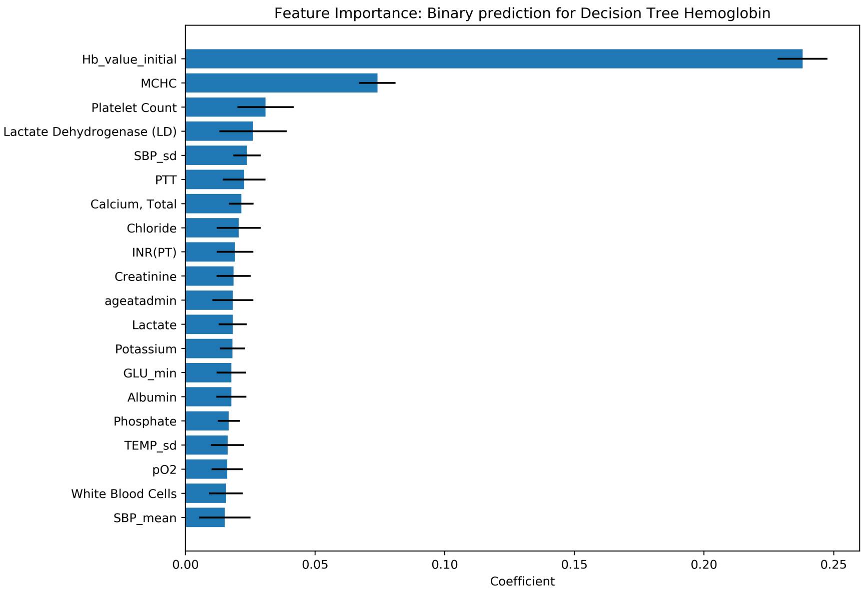

Supplementary Figure 19: **CART Feature Importance - Hemoglobin Classification**

8 Feature Importance by model - AKI Classification Task

(Shown Feature Importances are entropy-based, with the exception of logistic regression for which model coefficients are shown)

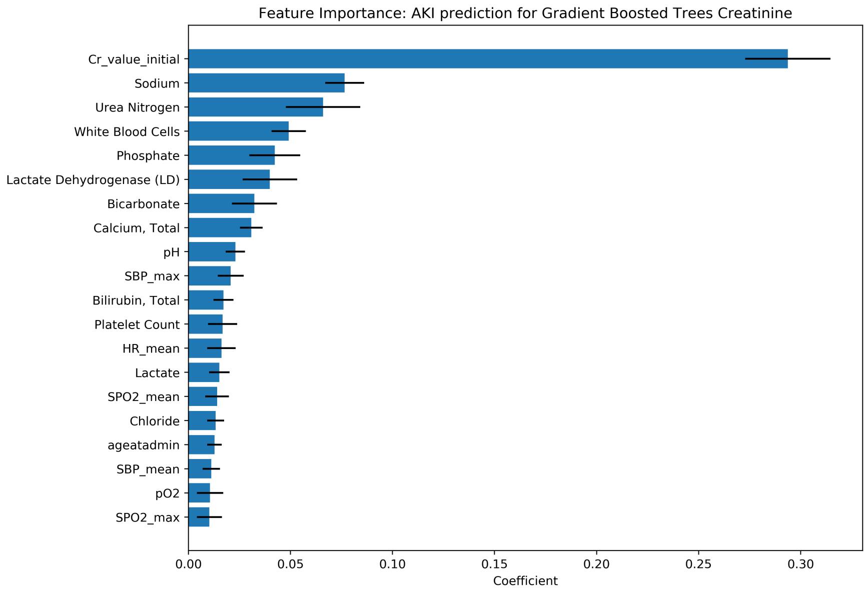

Supplementary Figure 20: **Gradient Boosted Trees Feature Importance - AKI Classification**

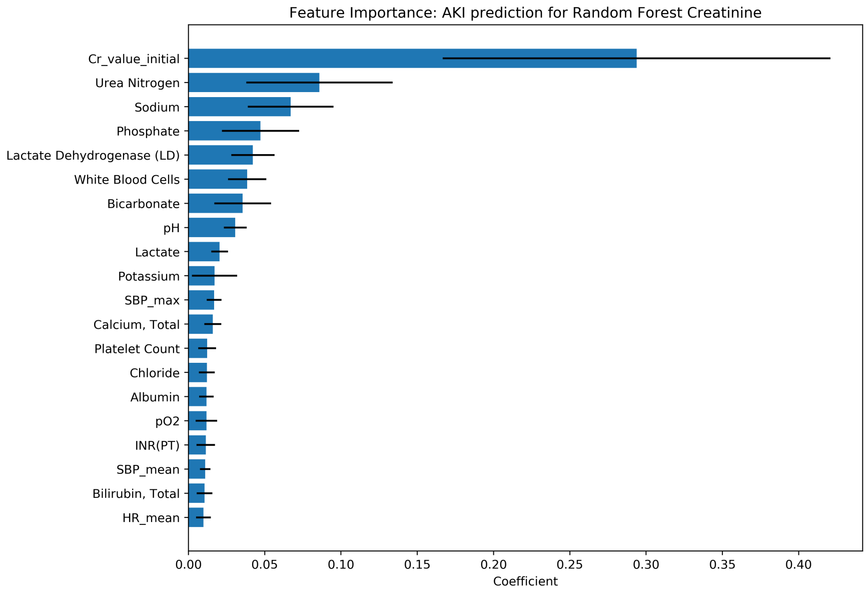

Supplementary Figure 21: **Random Forest Feature Importance - AKI Classification**

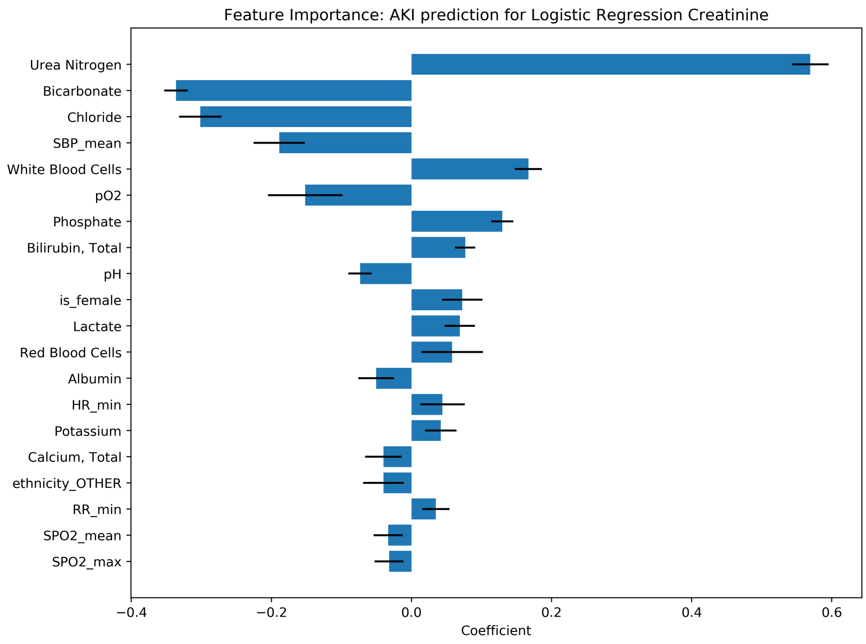

Supplementary Figure 22: **Logistic Regression Feature Importance - AKI Classification**

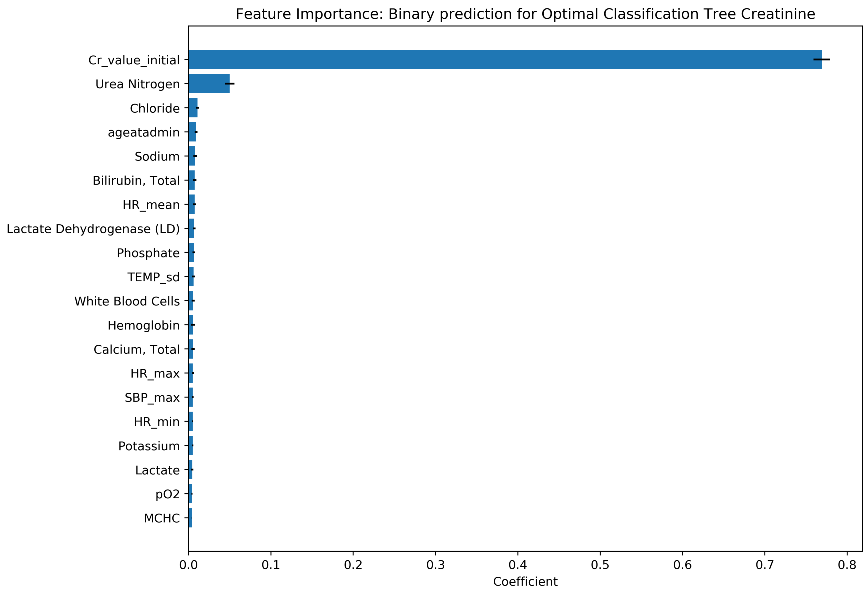

Supplementary Figure 23: **Optimal Classification Trees Feature Importance - AKI Classification**

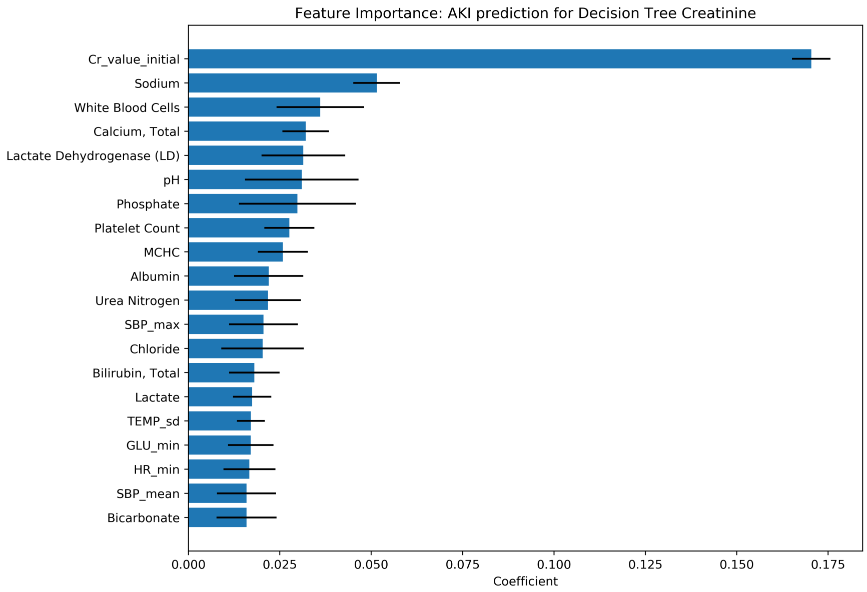

Supplementary Figure 24: **CART Feature Importance - AKI Classification**

9 Example: Data exploration prompted by Optimal Classificatin Tree splits

Non-intuitive interactions were identified in the optimal classification trees. For example, in one of the generated trees (shown below), glucose >450 mg/dl was predictive of normal renal function. Upon further investigation, a bimodal age distribution (shown below) was noted in the patients with glucose >450 mg/dl, suggesting that in some cases a markedly elevated glucose was simply a marker of younger age, which tends to be associated with better renal function. We suspect this finding was produced by a cohort of otherwise-healthy younger patients admitted for uncontrolled type I diabetes.

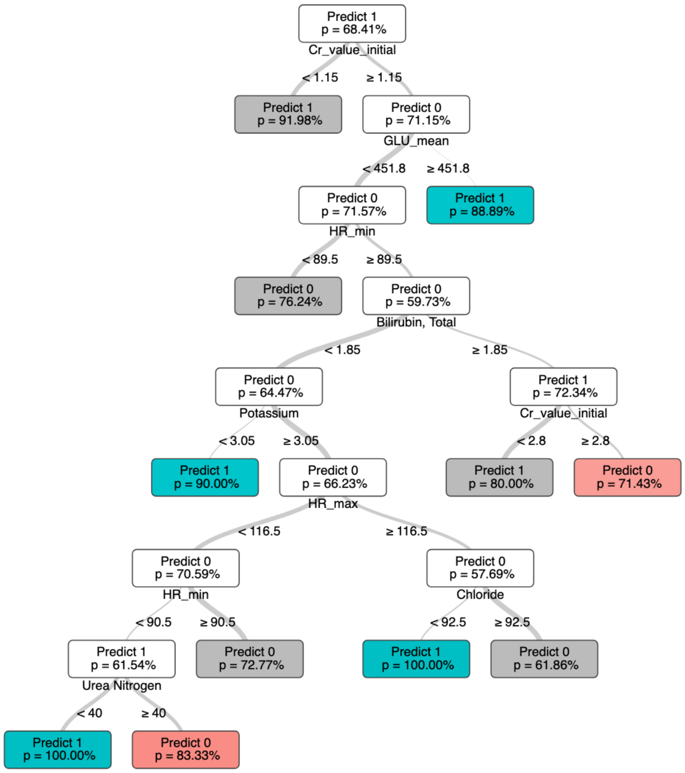

Supplementary Figure 25: Partial Optimal Classification Trees - Binary Creatinine Target (For Target Baseline Creatinine <1.3 mg/dL

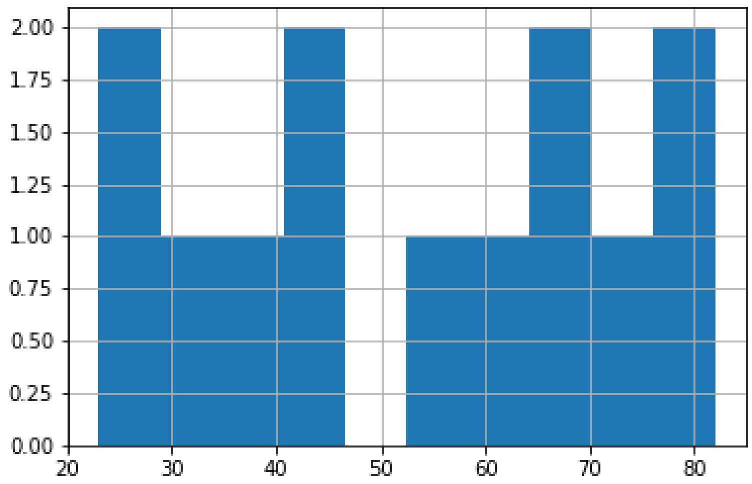

Supplementary Figure 26: Age distribution for creatnine cohort patients with mean glucose >450

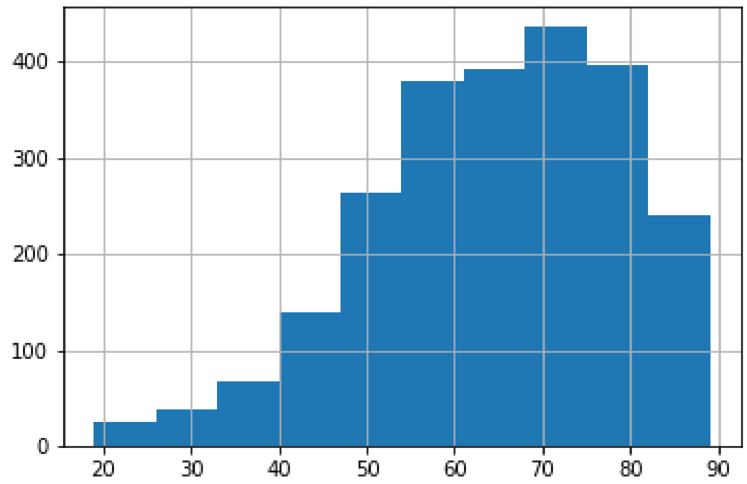

Supplementary Figure 27: Age distribution for creatinine cohort patients with mean glucose <450
